# Climate Change Reshapes the Habitat Suitability and Niche Stability of *Argentina anserina* (L.) Rydb. in Three Ethnic Prefectures of Sichuan Province Based on an Ensemble Model

**DOI:** 10.3390/biology15161357

**Published:** 2026-08-10

**Authors:** Yu Huang, Yong Huang, Yi Huang

**Affiliations:** 1 School of Computer and Network Security, Mianyang Teachers’ College, Mianyang 621000, China; huangyudemail@mtc.edu.cn (Y.H.); huangyong@mtc.edu.cn (Y.H.); 2 School of Geographical Sciences, China West Normal University, Nanchong 637009, China

**Keywords:** *Argentina anserina* (L.) Rydb., medicine–food homology, suitable habitat, ensemble modeling, western Sichuan Plateau

## Abstract

Climate change may substantially alter the habitat suitability and spatial stability of highland edible and medicinal plant resources. *Argentina anserina* (L.) Rydb. is an important wild resource plant in western China, but its potential distribution and future climate-change response in the three ethnic prefectures of Sichuan Province remain insufficiently understood. In this study, we used an ensemble species distribution modeling framework based on 31 spatially filtered occurrence records and 12 environmental predictors to evaluate the current and future habitat suitability of *A. anserina* under SSP126, SSP370, and SSP585 scenarios for the 2050s and 2090s. The ensemble model showed acceptable overall discrimination ability and was used to identify broad-scale suitability patterns. Mean diurnal range, precipitation of the driest quarter, isothermality, and topsoil pH were the main environmental predictors, indicating that thermal variability, dry-season moisture availability, and edaphic conditions jointly shaped habitat suitability. Under current conditions, the total suitable habitat area was predicted to be 15.57 × 10^4^ km^2^, accounting for 57.2% of the study region, with high-suitability habitats mainly concentrated in the northern, northwestern, and central areas. Future projections suggested an overall decline in suitable habitats, especially in high-suitability areas, although the magnitude varied among scenarios and periods. Spatial change and centroid migration analyses indicated that future suitable habitats may undergo contraction and westward or northwestward redistribution rather than uniform expansion. Niche overlap analysis further showed moderate to relatively high overlap between current and future environmental spaces, suggesting partial niche stability with scenario-dependent shifts. Given the limited field-based occurrence records, these findings should be interpreted as broad-scale spatial guidance rather than precise local predictions.

## Simple Summary

*Argentina anserina* is widely distributed across temperate and cold-temperate regions, but its underground organs develop into enlarged tuberous roots only in high-elevation environments such as the Qinghai–Tibet Plateau. These roots are locally known as “ginseng fruit.” In the three ethnic prefectures of western Sichuan, one of the species’ main production regions, *A. anserina* is an important source of food and income and contributes substantially to the livelihoods of highland farming and pastoral communities. However, climate change may alter where this species can grow in the future. In this study, we predicted the current and future suitable habitats of *A. anserina* in the three ethnic prefectures of Sichuan Province, China. We found that its distribution is mainly affected by daily temperature variation, precipitation during the driest season, temperature stability, and soil pH. At present, suitable habitats are mainly located in the northern, northwestern, and central parts of the study region. Under future climate scenarios, suitable habitats are expected to decrease overall, especially highly suitable areas. The center of suitable habitats may also shift westward or northwestward. These results suggest that climate change could reduce the stability of core habitats for *A. anserina*. Our findings can help guide field surveys, wild resource conservation, germplasm collection, and future cultivation planning for this valuable plant resource.

## 1. Introduction

Climate change is reshaping the distribution patterns, habitat suitability, and persistence of plant species worldwide. Rising temperatures, altered precipitation regimes, and increasing climatic variability can modify the environmental conditions required for plant survival, reproduction, and population maintenance, leading to habitat contraction, fragmentation, or geographic redistribution [1,2,3]. These effects are often more pronounced in mountainous and plateau regions, where steep elevational gradients, complex terrain, and strong local climatic heterogeneity create sensitive ecological transition zones [4]. For example, many alpine and subalpine plants have been reported to shift their suitable habitats toward higher elevations or cooler regions in response to warming, whereas species limited by moisture availability may experience habitat loss when dry-season precipitation declines [4,5]. Such changes are particularly important for wild edible and medicinal plants, because climate-driven habitat shifts may affect not only biodiversity conservation but also the sustainable use of regionally important biological resources.

*Argentina anserina* (L.) Rydb. is a perennial herb that is widely distributed across temperate and cold-temperate regions. However, its underground organs become markedly enlarged only in high-elevation habitats such as the Qinghai–Tibet Plateau, forming tuberous roots rich in starch, polysaccharides, flavonoids, and other bioactive compounds; these roots are therefore locally known as “ginseng fruit” [6]. As one of the species’ main production regions, the three ethnic prefectures of western Sichuan support its long-standing use as a traditional food and medicinal resource [6,7]. Its reported antioxidant, immunomodulatory, and stress-protective activities indicate potential for functional-food development and traditional medicinal products [7,8,9]. The species is also an important livelihood resource for highland farming and pastoral communities, contributing to food supplies, livestock feed, and household income. In western Sichuan and adjacent plateau-margin areas, *A. anserina* is often associated with alpine meadows, moist grasslands, river valleys, and other highland habitats [10,11]. Ganzi Tibetan Autonomous Prefecture, Aba Tibetan and Qiang Autonomous Prefecture, and Liangshan Yi Autonomous Prefecture are located along the eastern margin of the Qinghai–Tibet Plateau and within the transitional zone of the Hengduan Mountains. Their strong topographic relief and pronounced hydrothermal gradients provide diverse but environmentally sensitive habitats for highland plant resources. This region therefore provides an important setting for evaluating the habitat suitability and conservation potential of *A. anserina*.

Although the nutritional, pharmacological, physiological, and ethnobotanical characteristics of *A. anserina* have been widely investigated, its potential geographic distribution and climate-change response remain insufficiently understood. In particular, it is still unclear which environmental factors most strongly constrain its habitat suitability in complex mountain systems. For highland plants, habitat suitability is often controlled not only by annual mean temperature or precipitation, but also by short-term temperature fluctuation, seasonal water availability, soil chemical conditions, and terrain-related microclimates [12,13,14]. For instance, mean diurnal temperature range may influence physiological stress and growth stability [14], precipitation of the driest quarter may determine survival during seasonal water limitation [15], and soil pH may affect root-zone nutrient availability and underground organ development [16]. Huang et al. (2025) [17] recently modeled the potential distribution and cultivation zones of *A. anserina* in the Dadu River and Minjiang River basins. However, that analysis covered only approximately ten counties in Aba Prefecture, limiting its value for broad-scale regional planning and potentially increasing the risk of overfitting. By contrast, the present study encompasses dozens of counties across Ganzi, Aba, and Liangshan Prefectures. Although both studies relied on field-survey data, our broader spatial extent enables a more regional assessment and provides a more comprehensive theoretical basis for cultivation zoning across the western Sichuan Plateau. Nevertheless, suitable habitats of *A. anserina* and their responses to climate change have not yet been systematically assessed across all three prefectures.

Species distribution models provide an effective approach for predicting potential habitat suitability and assessing species responses to climate change. By linking known occurrence records with environmental variables, these models can identify key ecological constraints and project habitat suitability under current and future climate scenarios [18]. They have been widely applied to medicinal plants, endangered species, forest resources, and alpine vegetation [19,20,21,22]. For example, distribution modeling has been used to identify suitable cultivation regions for Panax notoginseng [23], predict future habitat changes in Coptis species [24], and support conservation planning for plateau plants under climate change. However, predictions based on a single algorithm may be influenced by model structure, occurrence-data limitations, pseudo-absence selection, and variable collinearity [25,26]. This uncertainty can be particularly important for species with limited occurrence records or for regions with complex mountainous environments. Ensemble modeling can integrate the outputs of multiple statistical and machine-learning algorithms, thereby reducing algorithm-specific bias and improving the robustness of broad-scale suitability assessment [25].

Accordingly, this study had three specific objectives: (1) to identify the key environmental factors constraining the current habitat suitability of *A. anserina*; (2) to project potential changes in suitable habitats in the 2050s and 2090s under three Shared Socioeconomic Pathways (SSP126, SSP370, and SSP585); and (3) to quantify future spatial dynamics, centroid migration, niche overlap, and niche breadth. We tested three corresponding hypotheses: (H1) habitat suitability is driven primarily by temperature variability and dry-season water availability rather than by annual mean climatic conditions; (H2) future climate change will cause a net contraction of suitable habitats, with the magnitude of contraction differing among emission scenarios; and (H3) climate-driven distributional changes will involve not only geographic displacement but also a partial shift in environmental niche space.

## 2. Materials and Methods

### 2.1. Occurrence Data

The target species in this study was A. anserina. Occurrence records were obtained from systematic field investigations conducted by the research team in the three ethnic prefectures of Sichuan Province, China, including Ganzi Tibetan Autonomous Prefecture, Aba Tibetan and Qiang Autonomous Prefecture, and Liangshan Yi Autonomous Prefecture, from July 2019 to November 2022. Details of the field surveys are provided in Text S1.

**Figure 1:**
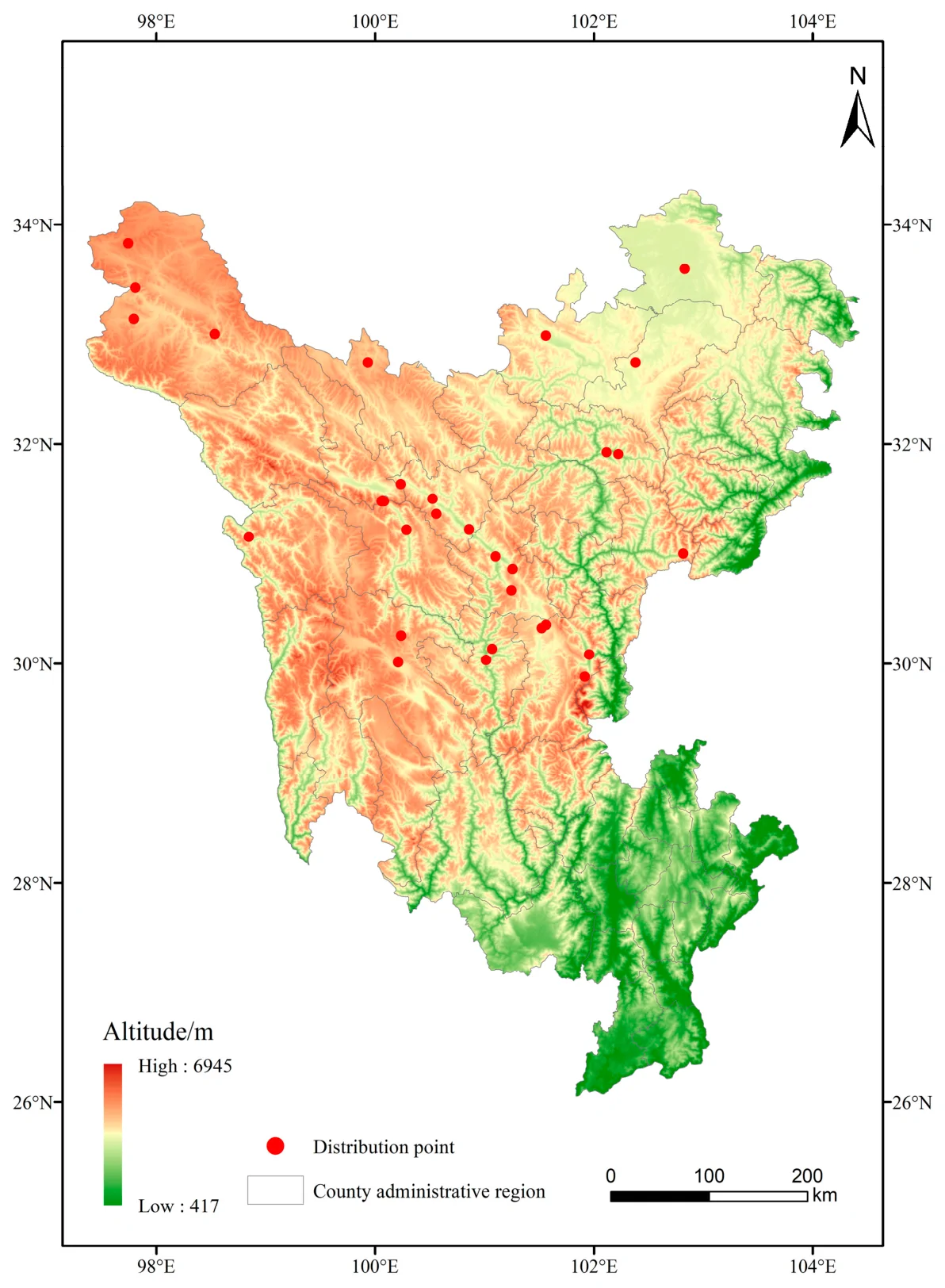
Study area and occurrence records of *A. anserina* in the three ethnic prefectures of Sichuan Province, China.

Before model calibration, the occurrence records were checked and cleaned by removing duplicated records, records without precise geographic coordinates, and points with obvious spatial errors. To reduce spatial sampling bias and spatial autocorrelation caused by clustered records, spatial thinning was performed using the R package spThin. A minimum distance threshold of 1 km was applied, allowing only one occurrence record to be retained within this distance [27]. In total, 66 original occurrence records were obtained, and 31 spatially filtered records were finally retained for subsequent species distribution modeling (Figure 1; Table S1). Although the final number of occurrence records was relatively limited, these records were obtained from field investigations and were spatially filtered to reduce clustering effects and spatial autocorrelation. For species distribution modeling, retaining fewer but spatially independent records is generally preferable to using clustered occurrence points that may overrepresent easily accessible or intensively surveyed areas [28,29]. Given the limited sample size, the modeling results were interpreted as broad-scale potential habitat suitability patterns rather than precise local distribution boundaries.

### 2.2. Environmental Variables and Selection

Environmental predictors were selected to represent the climatic, topographic, and edaphic conditions potentially affecting the distribution of *A. anserina*. A total of 26 environmental variables were initially considered, including 19 bioclimatic variables, three topographic variables, and four soil variables. Current and future climatic variables, topographic variables, and soil variables were obtained from publicly available spatial databases and standardized before model calibration. Detailed information on data sources, variable categories, spatial resolution, and preprocessing procedures is provided in Text S2.

**Figure 2:**
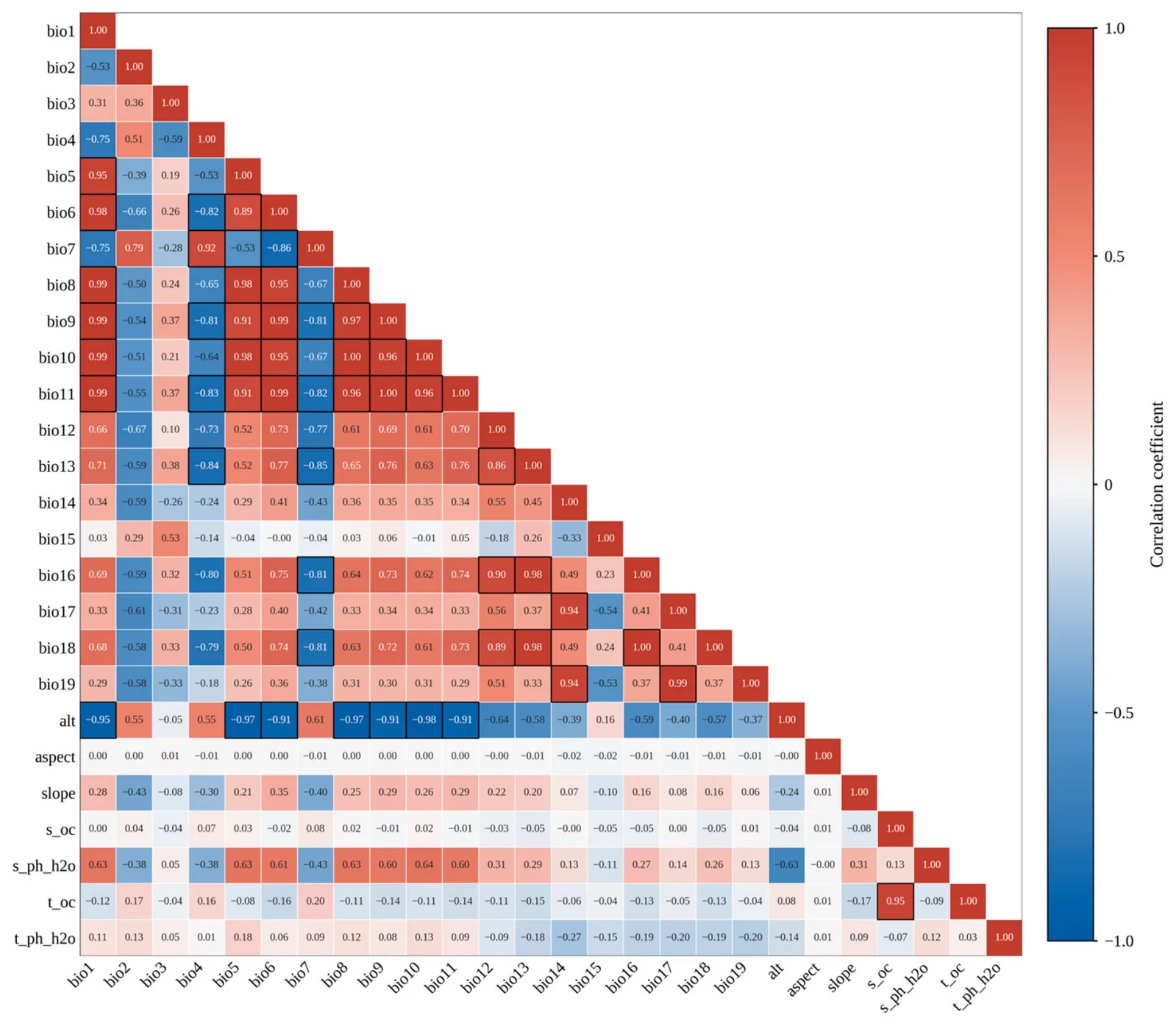
Pairwise correlation heatmap of candidate environmental predictors used for variable selection. The color gradient represents the correlation coefficient between variables. Predictors with |r| > 0.8 were considered highly correlated, and only the variable with the higher contribution rate was retained for model calibration.

To reduce model overfitting and multicollinearity, environmental variables were screened using a two-step procedure (Figure 2). First, all candidate variables and the spatially filtered occurrence records were imported into MaxEnt to estimate the relative contribution of each variable. Variables with zero contribution were removed because they provided little explanatory information for the preliminary model. Second, pairwise correlation analysis was performed for the remaining variables. When two variables showed high collinearity, defined as an absolute correlation coefficient greater than 0.8, only the variable with the higher MaxEnt contribution was retained [30,31,32]. This procedure reduced redundant environmental information while retaining predictors with stronger explanatory power for habitat suitability. This two-step approach combining preliminary contribution-based screening with correlation filtering is widely used for dimensionality reduction in species distribution modeling. It is particularly useful when occurrence records are limited, as it can reduce the risks of overfitting and multicollinearity. Nevertheless, variable-selection methods are not unique, and different screening strategies may produce slightly different predictor sets. The procedure adopted here is broadly consistent with comparable studies, thereby facilitating comparison across results.

After variable screening, 12 predictors were retained for subsequent Biomod2 modeling: seven bioclimatic variables, namely mean diurnal range (bio2), isothermality (bio3), temperature seasonality (bio4), maximum temperature of the warmest month (bio5), annual precipitation (bio12), precipitation seasonality (bio15), and precipitation of the driest quarter (bio17); two topographic variables, namely aspect and slope; and three soil variables, namely topsoil organic carbon (t_oc), topsoil pH in H_2_O (t_ph_h2o), and subsoil pH in H_2_O (s_ph_h2o) (Table S2).

### 2.3. Biomod2 Model Calibration and Ensemble Modeling

Species distribution modeling was performed using the Biomod2 package in R. To reduce the dependence of habitat suitability prediction on a single modeling algorithm, 12 individual algorithms were used, including artificial neural network (ANN), classification tree analysis (CTA), flexible discriminant analysis (FDA), generalized additive model (GAM), generalized boosted model (GBM), generalized linear model (GLM), multivariate adaptive regression splines (MARS), maximum entropy model (MaxEnt), maximum entropy model implemented in R (MAXNET), random forest with down-sampling (RFd), surface range envelope (SRE), and extreme gradient boosting (XGBOOST). These algorithms represent different statistical and machine-learning structures and can capture different forms of species–environment relationships.

Because reliable absence records were not available for *A. anserina*, pseudo-absence points were generated for model calibration. For each pseudo-absence dataset, 1000 pseudo-absence points were randomly generated within the study area. Future studies should consider M-area restriction, exclusion buffers, or target-group background sampling when larger samples or reliable dispersal data become available, and should test the sensitivity of model performance to the number of pseudo-absence points and the extent of the background area. Two pseudo-absence datasets were used to reduce the uncertainty associated with a single random background selection. For model evaluation, occurrence records were randomly divided into calibration and validation subsets, with 75% of the records used for model training and 25% used for testing. Each algorithm was repeated 10 times using random data partitioning to reduce stochastic effects and improve the stability of model evaluation.

Model performance was assessed using the true skill statistic (TSS), area under the receiver operating characteristic curve (AUC), and Cohen’s Kappa. TSS was used as the main criterion for selecting models for ensemble construction because it accounts for both sensitivity and specificity and is less affected by prevalence than some threshold-dependent metrics. Only individual models with TSS > 0.75 were retained for ensemble modeling, thereby excluding poorly performing models from the final prediction [33,34].

The final ensemble model was constructed using the weighted mean ensemble approach (EMwmean). In this approach, retained individual models were weighted according to their predictive performance, so that models with higher evaluation scores contributed more strongly to the final ensemble prediction. The EMwmean output was used as the final continuous habitat suitability map for subsequent current and future distribution projections.

### 2.4. Current and Future Habitat Suitability Prediction

The ensemble model was used to predict the habitat suitability of *A. anserina* under current and future climate conditions. For current habitat suitability modeling, the final predictor set consisted of 12 variables grouped into bioclimatic, topographic, and soil categories after screening based on MaxEnt variable contribution and multicollinearity analysis. For future projections, the calibrated model was transferred to future bioclimatic layers under SSP126, SSP370, and SSP585, representing low-, medium-high-, and high-emission pathways, respectively [35]. Two future periods were considered, namely the 2050s (2041–2060) and the 2090s (2081–2100). During model transfer, each future projection used the corresponding future bioclimatic layers together with the same topographic and soil layers used in the current model. This setting isolated the effect of climate change while avoiding unsupported assumptions about future changes in terrain and soil properties at the projection scale.

The continuous habitat suitability output ranged from 0 to 1, with higher values indicating more suitable environmental conditions for *A. anserina*. To ensure comparability among periods and scenarios, all suitability maps were classified using the same predefined thresholds: unsuitable habitat [0, 0.1), low-suitability habitat [0.1, 0.3), moderate-suitability habitat [0.3, 0.5), and high-suitability habitat [0.5, 1.0] [30,36]. The main advantage of applying fixed thresholds was that it ensured consistent classification of habitat suitability across all periods and scenarios. Although no universally accepted absolute standard exists for defining suitability classes, and alternative thresholds may affect the estimated area of each class, the overall spatial patterns and temporal trends are unlikely to change substantially. The area of each suitability class was calculated in a GIS environment for the current baseline and each future scenario, and these values were used to quantify temporal and scenario-dependent changes in potential suitable habitats.

### 2.5. Spatial Dynamics and Ecological Niche Analysis

To quantify spatial changes in habitat suitability of *A. anserina* under climate change, continuous suitability maps were reclassified into binary layers using the same thresholds defined in Section 2.4. Suitable habitats (including low-, moderate-, and high-suitability classes) and unsuitable habitats were used to generate spatial transition maps. Based on map overlay analysis, habitat change was categorized into stable suitable, newly suitable, lost, and stable unsuitable areas, and their corresponding areas were calculated in a GIS environment to assess habitat persistence and turnover under different scenarios.

Changes in environmental niche space were evaluated using occurrence records and climatic data for each period with the R package ecospat. Comparisons were conducted in a principal component analysis (PCA)-defined environmental space, with the entire study region used as the background. Kernel density functions with default smoothing parameters were used to estimate occurrence density and background environmental density. Schoener’s D and the niche-overlap index I were calculated to quantify overlap between current and future niches; both indices range from 0 to 1, with values closer to 1 indicating greater niche overlap [37]. Niche equivalency and similarity tests were each evaluated using 100 randomizations. Niche breadth in each period was quantified using Levins’ B1 (inverse concentration) and B2 (uncertainty) indices, both scaled from 0 to 1, with larger values indicating broader environmental tolerance. For centroid migration, the R package (v4.1.0) SDMTool (v1.1-21) was used to calculate the centroid coordinates of suitable habitats under each period and climate scenario. ArcGIS (v10.2) was then used to determine centroid longitude, latitude, and migration distance and to visualize migration pathways and trends.

## 3. Results

### 3.1. Model Performance and Reliability of Ensemble Prediction

**Figure 3:**
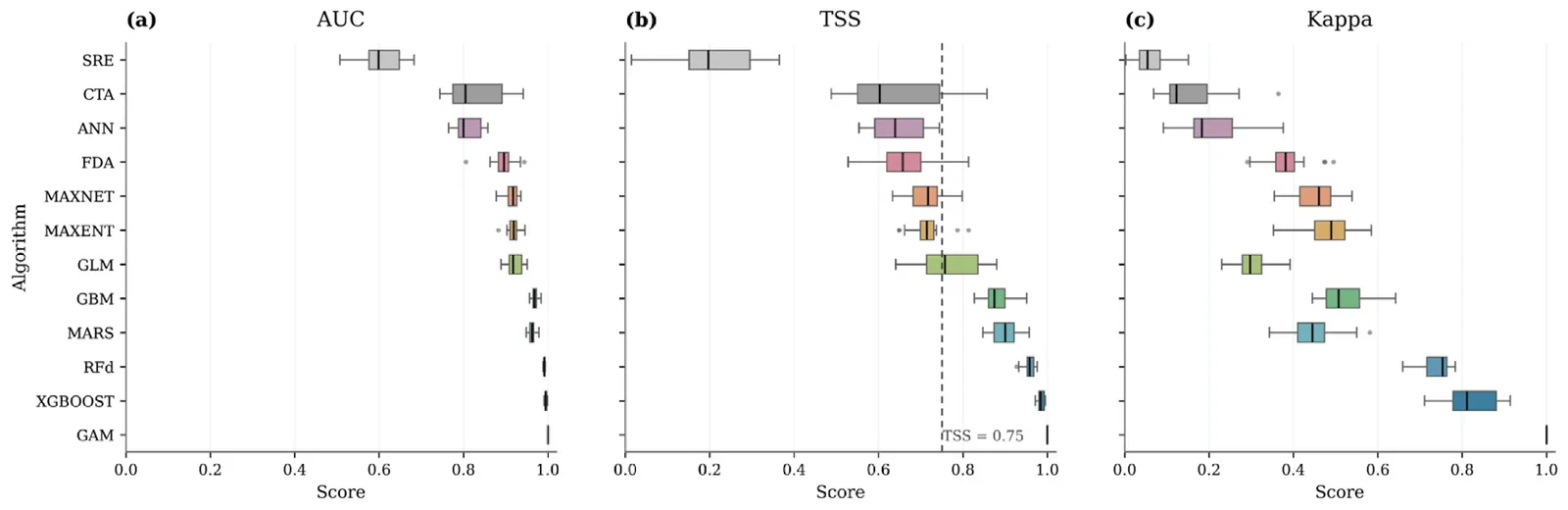
Model performance of single algorithms based on repeated calibration runs.

This section evaluates whether the ensemble model was sufficiently reliable to support the subsequent objectives. The predictive performance of the 12 single algorithms differed markedly across evaluation metrics (Figure 3; Table S3; Figure S1). Based on repeated calibration runs, most algorithms achieved high AUC values, indicating generally good discrimination between suitable and unsuitable habitats.

The EMwmean ensemble model produced an AUC value of 0.907, indicating acceptable overall discrimination ability. However, its threshold-dependent metrics were lower than those of the best-performing single algorithms, with a TSS value of 0.785 and a Kappa value of 0.625. Overall, the model evaluation results supported the use of ensemble modeling for subsequent habitat suitability prediction, but also highlighted the need to interpret the outputs cautiously given the limited number of occurrence records and the variation among individual algorithms.

### 3.2. Key Environmental Predictors Shaping Habitat Suitability

**Figure 4:**
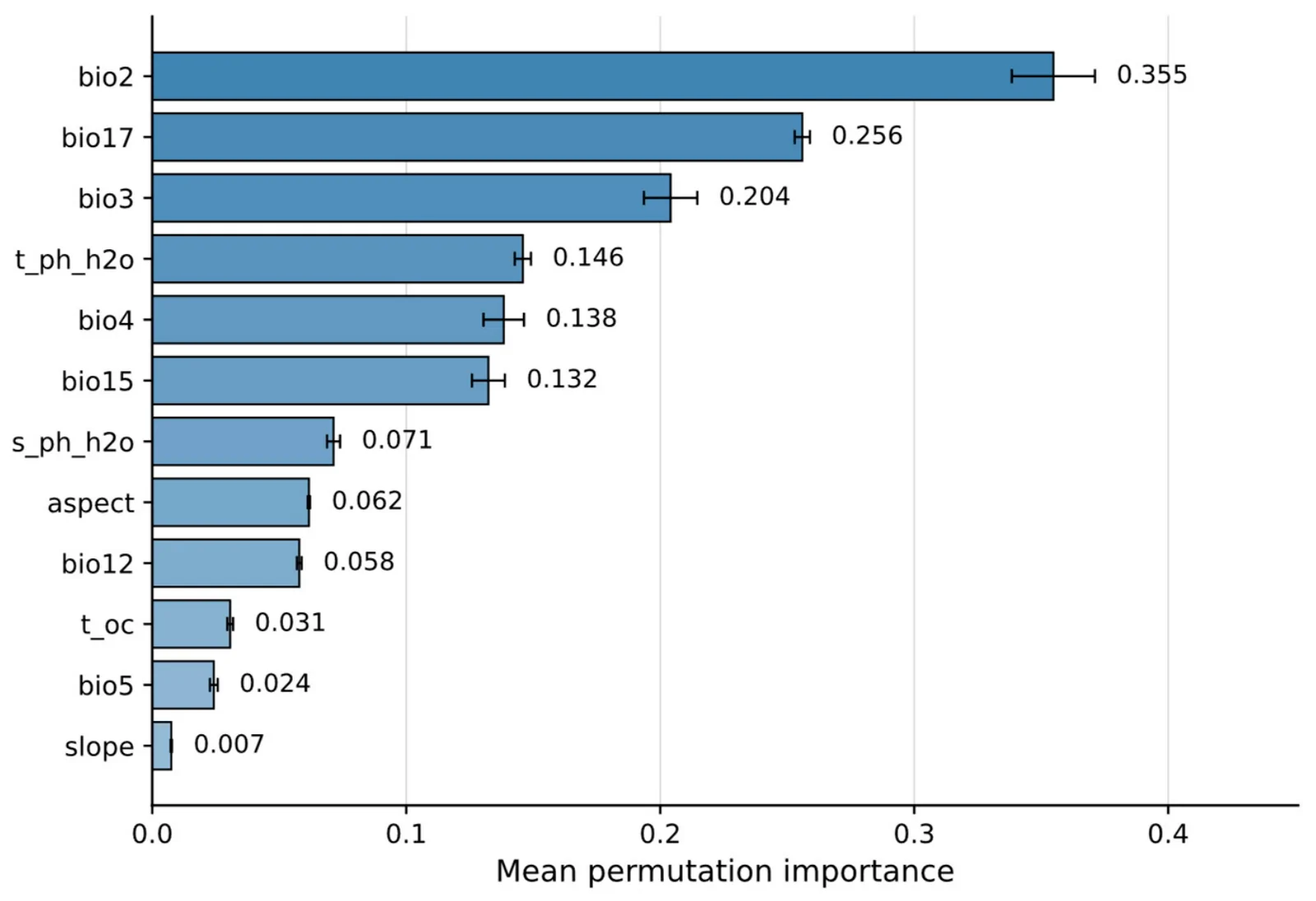
Relative importance of environmental predictors in the ensemble model. Bars indicate mean permutation importance values, and error bars represent standard deviations across repeated ensemble evaluations.

This section addresses objective 1 by identifying the key environmental drivers of current habitat suitability for *A. anserina* and testing hypothesis H1. The EMwmean ensemble model showed clear differences in the relative importance of the 12 retained environmental predictors (Figure 4).

Although their individual contributions were limited, these variables may still refine habitat suitability at local scales by modifying soil chemical conditions, drainage, solar radiation, and microclimatic environments [38]. Overall, the variable-importance results indicate that suitable habitats of *A. anserina* are mainly shaped by climatic variability and dry-season moisture availability, with soil pH acting as an important edaphic constraint. The response curves of the six most important predictors are shown in Figure S2.

### 3.3. Current Potential Suitable Habitats of A. anserina

**Figure 5:**
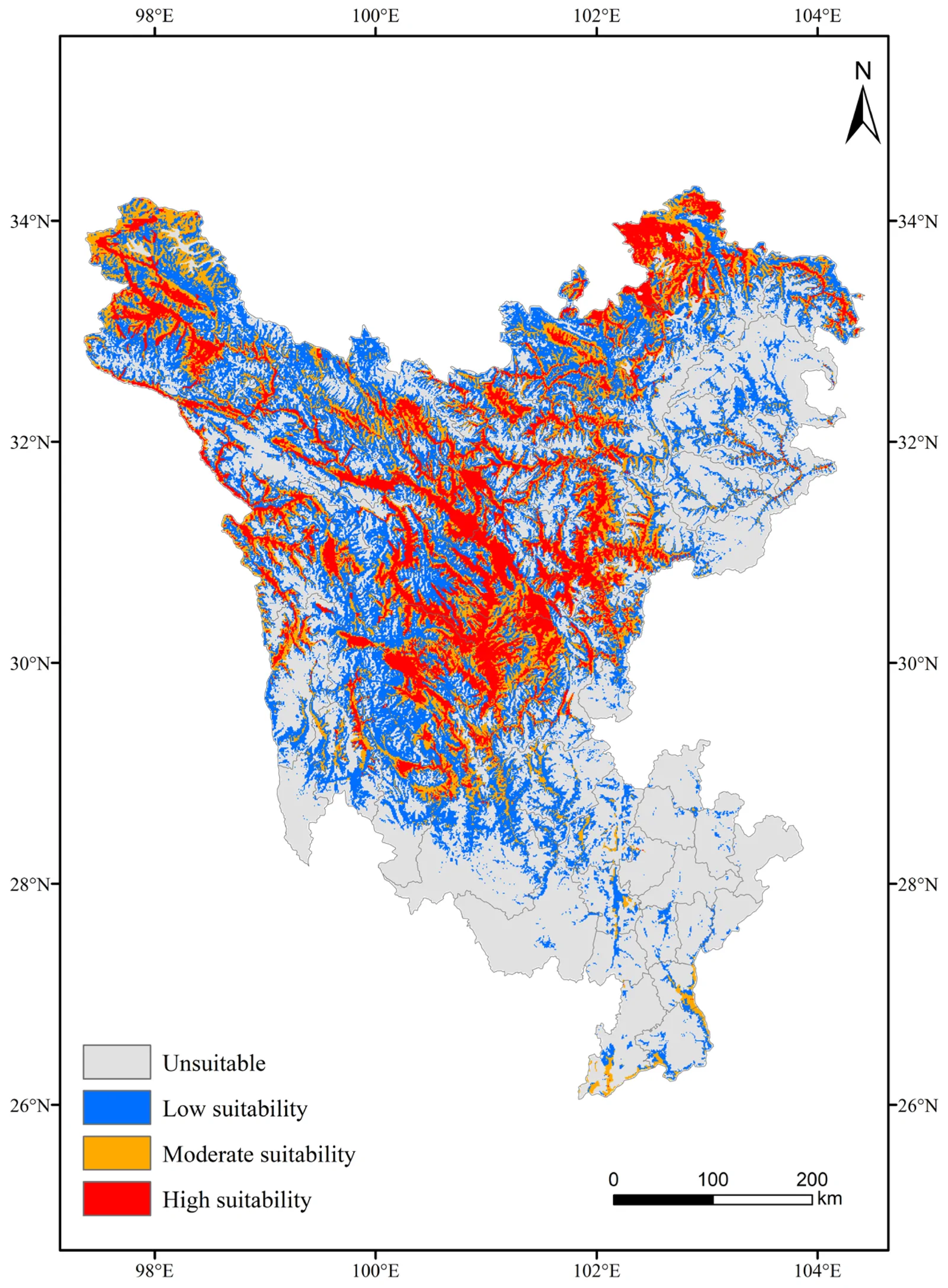
Current potential habitat suitability of *A. anserina* in the three ethnic prefectures of Sichuan Province, China. Habitat suitability was classified into unsuitable, low-suitability, moderate-suitability, and high-suitability habitats using predefined thresholds.

This section presents the potential suitable-habitat pattern of *A. anserina* under current climatic conditions, providing the baseline for objective 1 and the subsequent analyses. Under current environmental conditions, the EMwmean ensemble model predicted a total suitable habitat area of 15.57 × 10^4^ km^2^ for *A. anserina*, accounting for 57.2% of the study area (Figure 5; Table S4).

Spatially, suitable habitats were not evenly distributed across the three ethnic prefectures of Sichuan Province. High-suitability habitats were mainly concentrated in the northern, northwestern, and central parts of the study region, often forming belt-like and patchy distributions along mountain–valley systems. In contrast, the southern part of the study region was dominated by unsuitable and low-suitability habitats, with only scattered moderate- or high-suitability patches. This spatial pattern is consistent with the variable-importance results, which identified temperature variability, dry-season precipitation, and soil pH as the primary environmental drivers of habitat suitability for *A. anserina*. The coexistence of extensive low-suitability habitats and relatively concentrated high-suitability patches indicates that *A. anserina* has a broad potential distribution range but a more restricted core suitable area. Therefore, high- and moderate-suitability zones should be considered priority areas for field verification, wild resource conservation, and potential cultivation planning.

### 3.4. Future Changes in Habitat Suitability Under Climate Scenarios

**Figure 6:**
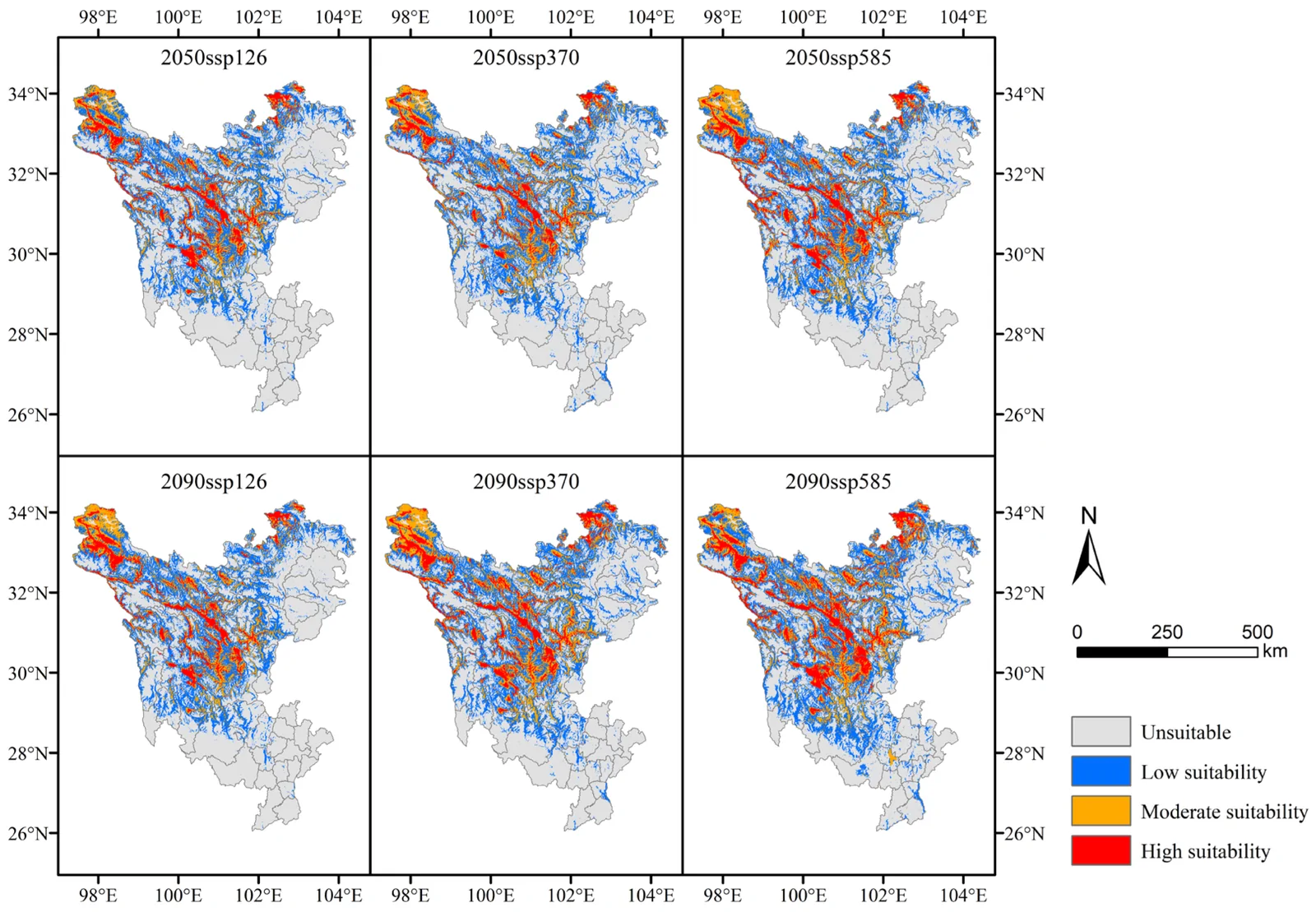
Future habitat suitability projections of *A. anserina* under different climate scenarios. Panels show projected habitat suitability in the 2050s and 2090s under SSP126, SSP370, and SSP585. Habitat suitability was classified into unsuitable, low-suitability, moderate-suitability, and high-suitability habitats.

This section addresses objective 2 by projecting changes in habitat suitability in the 2050s and 2090s under different climate scenarios and testing hypothesis H2. Future projections indicated that the potential suitable habitats of *A. anserina* generally decreased compared with the current baseline, although the magnitude of change varied among climate scenarios and time periods (Figure 6; Table 1; Figure S3).

Across future scenarios, unsuitable areas were consistently larger than under the current climate, increasing from 11.65 × 10^4^ km^2^ at present to 13.25–15.86 × 10^4^ km^2^ in future projections. However, the 2090s SSP585 scenario showed the largest future suitable area among all projected scenarios, mainly due to increases in low- and high-suitability habitats. This indicates that future climate change may not simply cause a uniform loss of suitable habitats, but may drive suitability-level shifts and spatial redistribution within the study region.

### 3.5. Spatial Dynamics of Habitat Suitability and Changes in Environmental Niche Space

**Figure 7:**
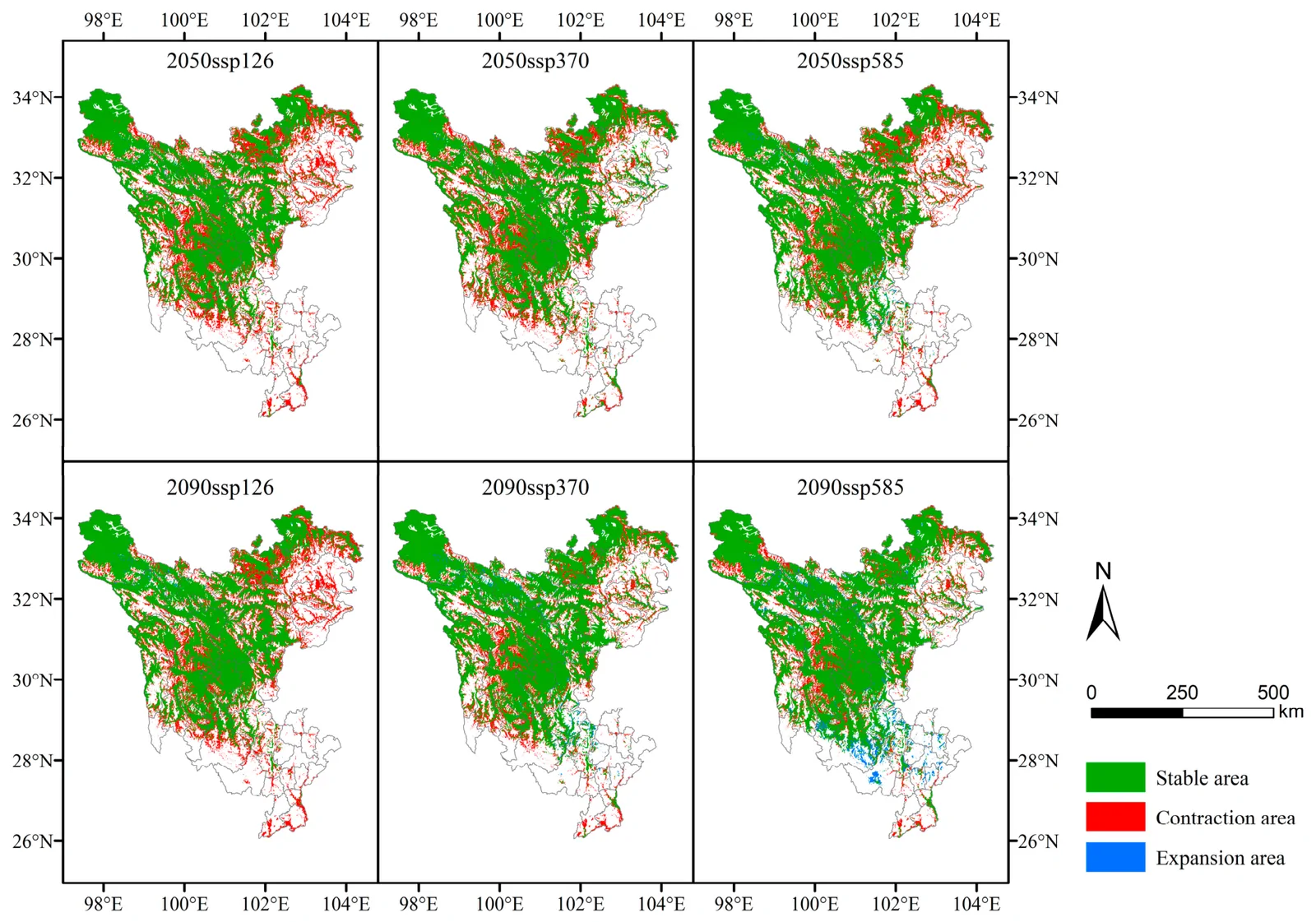
Spatial changes in suitable habitats of *A. anserina* under future climate scenarios.

This section addresses objective 3 by quantifying future spatial dynamics, centroid migration, and changes in environmental niche space and by testing hypothesis H3. Future projections indicated a consistent decline in the total suitable habitat area of *A. anserina* across all six future climate scenarios, with the most pronounced reduction occurring in the high-suitability class. Relative to the current total suitable area of 15.57 × 10^4^ km^2^, the decline was particularly marked for high-suitability habitats (Figure 7; Figure S4; Table S5).

Spatially, projected habitat changes were characterized primarily by contraction and reorganization rather than uniform range expansion. Across all scenarios, the area of lost suitable habitat substantially exceeded that of newly suitable habitat.

**Figure 8:**
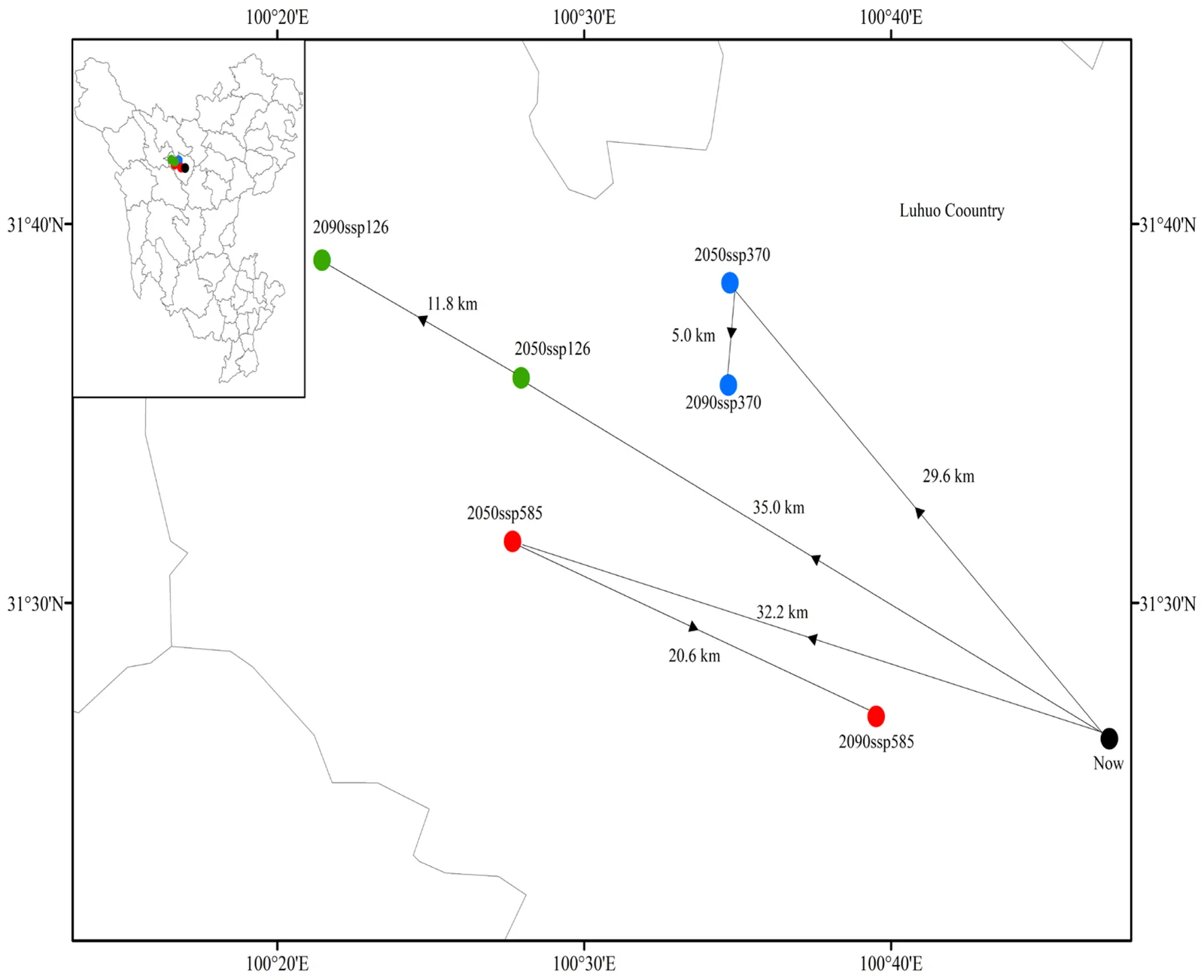
Centroid shifts in potential suitable habitats under future climate scenarios.

Centroid analysis revealed directional shifts in the geographic center of suitable habitats under future climate scenarios (Figure 8; Table S6). Under current conditions, the suitable-habitat centroid was located at approximately 100.785° E and 31.440° N. Future centroids generally shifted westward or northwestward relative to the current centroid, although migration distance varied among scenarios and periods. These results indicate that climate change may alter the spatial center of suitable habitats, but the magnitude and direction of movement will depend on the scenario and time period.

**Figure 9:**
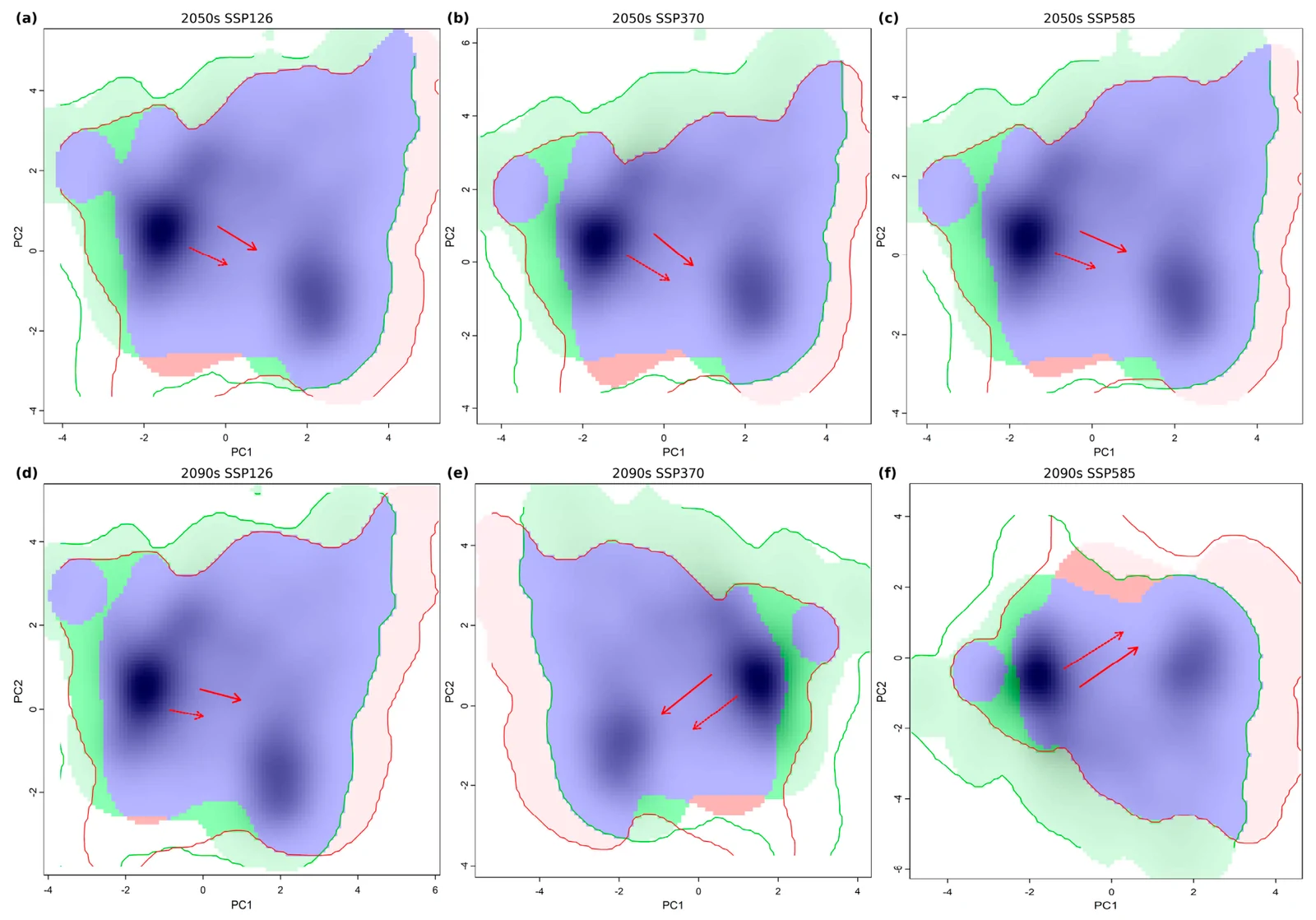
Ecological niche overlap between current and future climate scenarios for *A. anserina*. Panels represent niche overlap under (**a**) 2050s SSP126, (**b**) 2050s SSP370, (**c**) 2050s SSP585, (**d**) 2090s SSP126, (**e**) 2090s SSP370, and (**f**) 2090s SSP585.The solid contours encompass 100% of the available environmental space, whereas the dashed contours represent 50% of the space. The movement of the climatic ecological niche of *A. anserina* (solid line) and its background range center (dashed line) between the two extents are illustrated by red arrows.

Niche-overlap analysis showed that Schoener’s D ranged from 0.534 to 0.702 and the niche-overlap index I ranged from 0.634 to 0.796 across future scenarios (Figure 9; Table S7). Overlap was relatively greater under SSP126 but decreased under long-term and high-emission scenarios, reaching its lowest value under SSP585 in the 2090s. This pattern was consistent with the projected reduction in suitable habitats and contraction of high-suitability areas (Figure 6; Table 1).

## 4. Discussion

### 4.1. Reliability and Uncertainty of Ensemble Habitat Suitability Modeling

The reliability of species distribution models is typically influenced by the following factors: the quality and quantity of distribution records, the selection of environmental variables, pseudo-absence sampling and selection, threshold selection, and differences among modeling algorithms [39,40,41]. These sources of uncertainty are particularly important for species with limited distribution records or for studies conducted in mountainous areas with high environmental heterogeneity. In the present study, the 12 individual algorithms differed markedly in performance, supporting the use of an ensemble framework for modeling mountain species. At the same time, the difficulty of some algorithms, including SRE and ANN, in converging with only 31 occurrence records indicates that model evaluation may have been strongly influenced by pseudo-absence sampling and sample size. Thus, although ensemble modeling can reduce algorithm-specific uncertainty, the projections should be regarded as preliminary, broad-scale estimates rather than precise confirmation of the species’ distribution.

The ensemble model demonstrated acceptable overall discriminatory power, with an AUC value of 0.907, indicating that it can distinguish between generally suitable and unsuitable habitats. Previous studies indicate that validation of species distribution models for alpine medicinal plants should integrate AUC, TSS, and independent occurrence data and should interpret threshold-dependent metrics cautiously [42,43]. Given these limitations, the habitat-suitability maps produced here are best used to prioritize areas for field validation and resource surveys rather than treated as direct representations of the species’ realized distribution. The predicted high-suitability areas broadly coincided with known collection sites and with alpine meadows, moist grasslands, and river-valley habitats described in the literature, providing indirect support for the model’s broad-scale indicative value. Future studies should test and calibrate the predicted suitable areas using independent field plots, germplasm-resource surveys, and local microclimate observations, while also evaluating the influence of sampling bias on model inference.

### 4.2. Environmental Controls on Habitat Suitability of A. anserina

The variable-importance results indicated that habitat suitability of *A. anserina* was primarily driven by climatic variability and seasonal water limitation. This pattern is consistent with previous studies demonstrating that temperature variability and dry-season moisture availability are often more influential than annual mean climate in shaping species distributions [44,45]. In alpine ecosystems, such variables are particularly important because plant establishment and perennial growth are strongly constrained by daily thermal fluctuations and seasonal drought stress [46]. As a perennial herb, *A. anserina* develops enlarged tuberous roots rich in starch and polysaccharides that serve as important edible and medicinal storage organs [6]. The species commonly occurs at elevations of 3000–4200 m in alpine meadows, floodplains, and moist grasslands on gentle slopes, where diurnal temperature variation is pronounced and wet and dry seasons are distinct [6]. Moderate day–night temperature differences may therefore promote carbohydrate accumulation in underground organs, whereas dry-season water availability may directly affect sprouting and perennial survival.

The high contribution of bio2 suggests that daily temperature fluctuation may be a key constraint on the suitable habitats of *A. anserina*. In mountainous regions, strong diurnal temperature variation reflects the combined effects of elevation, solar radiation, and nighttime cooling. Moderate temperature differences may favor carbohydrate accumulation in underground organs, whereas excessive fluctuation may increase physiological stress and reduce population persistence [17,46]. Similarly, the importance of bio17 indicates that precipitation during the driest quarter is critical for maintaining soil moisture during seasonally water-limited periods. *A. anserina* has a sparse, shallow rhizome and root system and, at high elevations, often develops a single enlarged tuberous root. It may therefore be sensitive to changes in surface-soil moisture. Insufficient dry-season precipitation could cause wilting or even plant mortality, consistent with local Tibetan observations of reduced harvests in drought years. Compared with annual precipitation, dry-season precipitation may better explain habitat suitability in plateau-margin regions, where water availability is unevenly distributed across seasons [47,48].

Soil pH also contributed meaningfully to habitat suitability, indicating that edaphic conditions may influence the establishment and growth of *A. anserina* through effects on nutrient availability, root-zone chemical environment, and underground organ development [49]. Although variables such as slope, aspect, annual precipitation, topsoil organic carbon, and maximum temperature of the warmest month showed lower individual importance, they may still regulate habitat suitability at local scales by modifying drainage, solar radiation, soil moisture retention, and microclimatic conditions [50,51]. Overall, the distribution of *A. anserina* in the study region appears to be determined by the combined effects of thermal variability, dry-season moisture supply, and soil pH, with topographic factors acting as secondary modifiers. Therefore, areas with moderate diurnal temperature variation, sufficient dry-season precipitation, and suitable topsoil pH should be prioritized for field verification, germplasm collection, and potential cultivation planning.

### 4.3. Climate-Driven Habitat Redistribution and Niche Stability of A. anserina

Climate change may not only reduce the total extent of potential habitats but also weaken the spatial continuity and stability of core suitable areas. For resource plants with relatively specific habitat requirements, the loss of high-suitability habitats is more ecologically important than changes in low-suitability areas [52], because these core habitats are more likely to support stable populations, long-term regeneration, and high-quality germplasm resources.

The greater loss than gain of suitable habitats indicates that future environmental conditions may exceed the tolerance range of *A. anserina* in parts of its current suitable habitats. This pattern is consistent with the environmental-control results, which showed that the species is sensitive to temperature variability and dry-season water availability [10,17]. In mountain systems, warming may alter local hydrothermal balance, increase seasonal water stress, or shift suitable conditions toward higher-elevation and more humid microhabitats. However, the model did not account for local habitat modification caused by grazing, harvesting, or road construction, which may further reduce the area of habitat that is actually available. From a resource-management perspective, dynamic monitoring should therefore prioritize areas that are projected to be lost but where collection still occurs.

The projected westward or northwestward centroid shift may reflect the movement of suitable hydrothermal combinations toward areas with cooler conditions, stronger topographic buffering, or more favorable dry-season moisture availability. However, centroid movement should not be interpreted as direct species migration, because actual range shifts may be constrained by dispersal limitation, habitat fragmentation, land use, harvesting pressure, and local population dynamics. The projected redistribution should therefore be regarded as a spatial signal of changing habitat suitability rather than evidence of realized population movement. In practical resource-use planning, the direction of centroid movement can provide broad guidance for future surveys, germplasm collection, and resource utilization, but candidate areas must be screened for field accessibility and current land use. Stable suitable habitats should be prioritized for in situ conservation and sustainable use, whereas lost and marginal habitats require enhanced monitoring to limit further erosion of germplasm resources under climate change.

Niche-overlap results further indicated that the projected redistribution of *A. anserina* was not simply a geographic shift but also involved changes in environmental suitability space. The moderate to relatively high niche overlap suggests that *A. anserina* may retain part of its current environmental requirements under future climate conditions, but its niche stability is clearly scenario-dependent [37]. The relatively higher overlap under SSP126 indicates that low-emission pathways may maintain environmental conditions more similar to the current niche. In contrast, the lower overlap under SSP585, especially by the 2090s, implies a stronger departure from the current suitability space [53]. Although some areas may remain suitable or become newly suitable, the environmental combinations supporting high suitability are likely to change, particularly under stronger climate forcing. Therefore, the future response of *A. anserina* should not be interpreted as a simple range shift within a fully conserved niche. Instead, climate change may simultaneously reduce core suitable habitats, alter suitability levels, and drive partial redistribution toward areas with more favorable hydrothermal and edaphic conditions [37,54]. The westward or northwestward centroid shifts further support this interpretation (Table S6), suggesting that future suitable environments may move toward regions with stronger topographic buffering and more suitable seasonal moisture conditions [55,56].

## 5. Conclusions

This study evaluated the current and future habitat suitability of *A. anserina* across the three ethnic prefectures of Sichuan Province. Its potential distribution was jointly constrained by mean diurnal range, precipitation of the driest quarter, isothermality, and topsoil pH, indicating that thermal variability, dry-season water availability, and edaphic conditions collectively shape habitat suitability. Under future climate scenarios, total suitable habitat generally declined, with particularly pronounced losses in high-suitability areas, suggesting that climate change may weaken the stability of core habitats. Suitable habitats were also projected to shift westward or northwestward. Given the limited sample size, these findings should be interpreted as broad-scale spatial guidance rather than precise local predictions. They can nevertheless inform field validation, wild-resource conservation, germplasm collection, and potential cultivation planning for *A. anserina* in highland ethnic regions.

## Figures and Tables

**Table 1:** Predicted habitat suitability area of *A. anserina* under current and future climate scenarios (10^4^ km^2^).

Period/Scenario	Unsuitable	Low Suitability	Moderate Suitability	High Suitability	Total Suitable
Current	11.65	7.92	3.97	3.68	15.57
2050s SSP126	15.86	6.62	2.96	1.78	11.36
2050s SSP370	15.24	7.13	3.32	1.52	11.97
2050s SSP585	14.79	6.98	3.52	1.94	12.44
2090s SSP126	15.75	6.69	3.06	1.73	11.48
2090s SSP370	14.12	7.07	3.84	2.19	13.10
2090s SSP585	13.25	7.62	3.84	2.52	13.98

## Data Availability

The occurrence data and model outputs generated in this study are available from the corresponding author upon reasonable request. Environmental variables were obtained from the publicly available databases described in the Supplementary Materials.

## Supplementary Materials

The following supporting information can be downloaded at: https://www.mdpi.com/article/10.3390/biology15161357/s1. Text S1: Field occurrence records and spatial filtering; Text S2: Environmental predictors and data preprocessing; Figure S1: TSS-based ranking of single algorithms used for species distribution modeling; Figure S2: Response curves of the six most important environmental predictors in the EMwmean ensemble model; Figure S3: Area composition of habitat suitability classes for *A. anserina* under current and future climate scenarios; Figure S4: Area composition of stable suitable, lost suitable, and newly suitable habitats under future climate scenarios; Table S1: Latitude and longitude of species distributions; Table S2: Environmental predictors retained for model calibration; Table S3: Model evaluation metrics of single algorithms and the EMwmean ensemble model; Table S4: Current habitat suitability area of *A. anserina* in the study region; Table S5: Areas of stable suitable, lost suitable, and newly suitable habitats under future climate scenarios; Table S6: Spatial dynamics and centroid migration of suitable habitats of *A. anserina* under future climate scenarios; Table S7: Niche overlap indices between current and future climate scenarios.
