# Supplementary material for: Climate Change Reshapes the Habitat Suitability and Niche Stability of Argentina anserina (L.) Rydb. in Three Ethnic Prefectures of Sichuan Province Based on an Ensemble Model
Source: Biology (Basel). 2026 Aug 10;15(16):1357. doi: 10.3390/biology15161357 (PMC13509757; doi:10.3390/biology15161357)
Supplement: Supplementary file 1 [file biology-15-01357-s001.zip › biology-4451507-supplementary.pdf]

Supporting materials

Climate Change Reshapes the Habitat Suitability and Niche Stability of *Argentina anserina* (L.) Rydb. in three ethnic prefectures of Sichuan Province

Yu Huang[1], Yong Huang[1], Yi Huang[2*]

[1] School of Computer and Network Security, Mianyang Normal University, Mianyang 621000, China; huangyudemail@mtc.edu.cn (Y.H. (Yu Huang)); huangyong@mtc.edu.cn (Y.H. (Yong Huang))

[2] School of Geographical Sciences, China West Normal University, Nanchong 637009, China; hyhy1232021@163.com (Y.H. (Yi Huang))

* Correspondence: hyhy1232021@163.com (Y.H.)

Text S1. Field occurrence records and spatial filtering

Occurrence records of *A. anserina* were obtained exclusively from field investigations conducted in the three ethnic prefectures of Sichuan Province, China, including Ganzi Tibetan Autonomous Prefecture, Aba Tibetan and Qiang Autonomous Prefecture, and Liangshan Yi Autonomous Prefecture. No records from literature, herbarium databases, online biodiversity platforms, or previously published distribution datasets were used for model calibration.

During field surveys, the presence of *A. anserina* was confirmed by direct observation of living plants in natural habitats. Geographic coordinates were recorded in decimal degrees using GPS-enabled field devices, and related field information, such as administrative location, habitat type, and elevation when available, was also documented for verification.

A total of 66 original field occurrence records were obtained. Before modeling, duplicated records, records without precise coordinates, and points with obvious spatial errors were removed. All coordinates were checked in a GIS environment to ensure that they were located within the study region.

To reduce spatial sampling bias and autocorrelation caused by clustered records, spatial thinning was performed using the R package spThin. A minimum distance threshold of 1 km was applied, allowing only one occurrence record to be retained within this distance. After data cleaning and spatial thinning, 31 occurrence records were retained as the final presence dataset for Biomod2 model calibration and habitat suitability prediction.

Text S2. Environmental predictors and data preprocessing

Environmental predictors used in this study were selected to characterize the climatic, topographic, and edaphic conditions potentially influencing the habitat suitability of *A. anserina*. A total of 26 candidate environmental variables were initially considered, including 19 bioclimatic variables, three topographic variables, and four soil variables. The 19 bioclimatic variables (bio1–bio19) were obtained from the WorldClim version 2.1 database (http://worldclim.org/data/index.html; accessed on 10 May 2026), with a spatial resolution of 30 arc-seconds (approximately 1 km) and a baseline climate period of 1970–2000. Future bioclimatic variables were derived from the downscaled CMIP6 dataset, using the BCC-CSM2-MR global climate model under three Shared Socioeconomic Pathway (SSP) scenarios: SSP126 (low-emission scenario), SSP370 (intermediate-to-high emission scenario), and SSP585 (high-emission scenario). Two future periods were considered: the 2050s and 2090s. Topographic variables included elevation, slope, and aspect. Elevation was obtained from the WorldClim elevation layer at the same spatial resolution as the bioclimatic variables, and slope and aspect were derived from the elevation layer in a GIS environment. Soil variables included topsoil organic carbon (t_oc), subsoil organic carbon (s_oc), topsoil pH in H_2O (t_ph_h2o), and subsoil pH in H_2O (s_ph_h2o). Soil data were obtained from the Harmonized World Soil Database (HWSD v1.2), with the official data source available at https://www.fao.org/soils-portal/soil-survey/soil-maps-and-databases/harmonized-world-soil-database-v12/en/ (accessed on 10 May 2026). All environmental raster layers were clipped to the boundary of the three ethnic prefectures of Sichuan Province, including Ganzi Tibetan Autonomous Prefecture, Aba Tibetan and Qiang Autonomous Prefecture, and Liangshan Yi Autonomous Prefecture. The raster layers were processed to ensure a consistent coordinate system, spatial extent, and spatial resolution before model calibration. For future projections, only climatic variables were replaced by corresponding future climate layers, whereas topographic and soil variables were kept constant because they are generally assumed to remain stable over the projection periods. Candidate variables were further screened before Biomod2 modeling. Variables with zero contribution in the preliminary MaxEnt model were removed, and the remaining variables were subjected to pairwise correlation analysis. When two variables were highly correlated, only the variable with the higher MaxEnt contribution was retained. This

screening strategy was used to balance preliminary explanatory power with multicollinearity control, while retaining predictors with stronger ecological relevance to habitat suitability. The final predictor set contained 12 variables used for ensemble modeling.

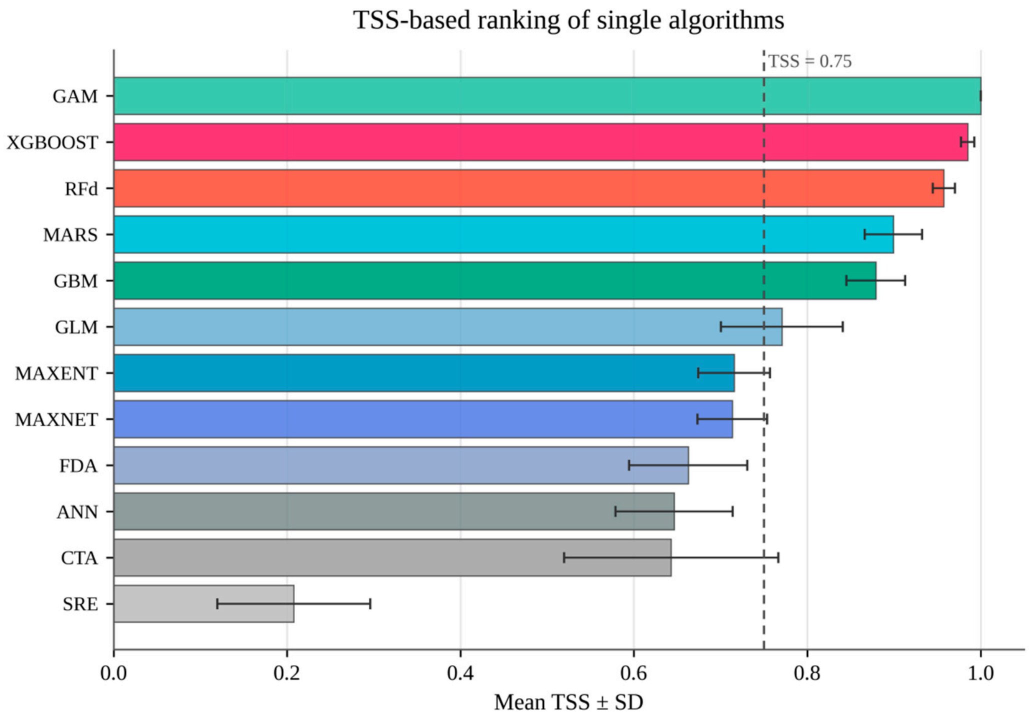

Figure S1. TSS-based ranking of single algorithms used for species distribution modeling. Bars indicate mean TSS values, and error bars represent standard deviations across repeated calibration runs. The dashed line indicates the TSS threshold of 0.75.

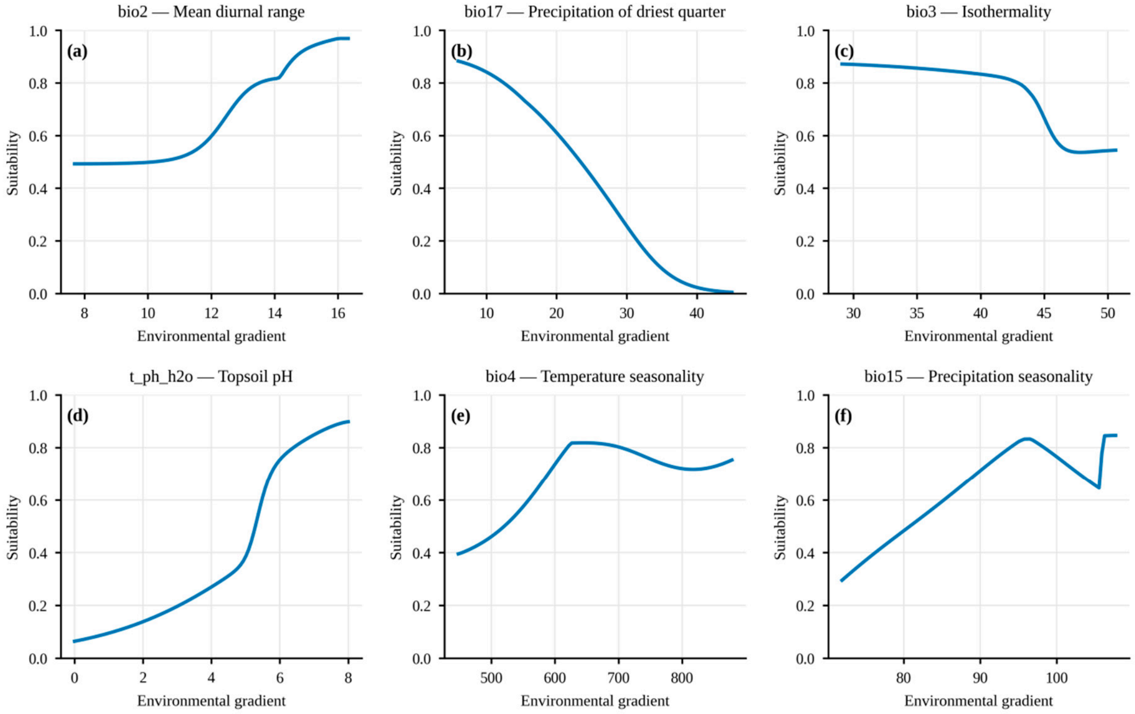

Figure S2. Response curves of the six most important environmental predictors in the EMwmean ensemble model. Curves show marginal changes in predicted habitat suitability along each environmental gradient.

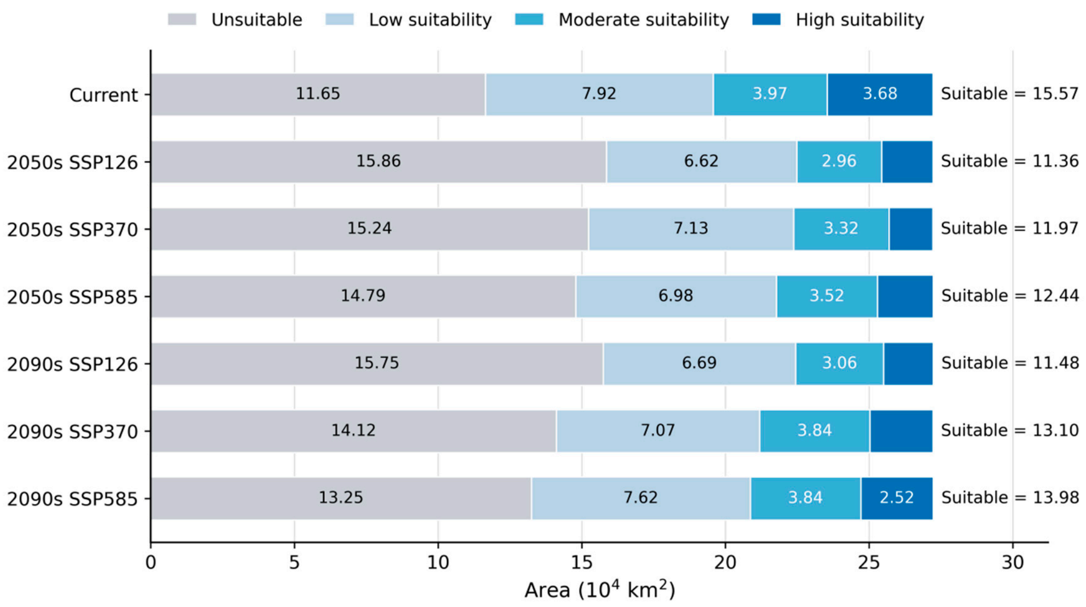

Figure S3. Area composition of habitat suitability classes for *A. anserina* under current and future climate scenarios. Values above bars indicate the total suitable habitat area.

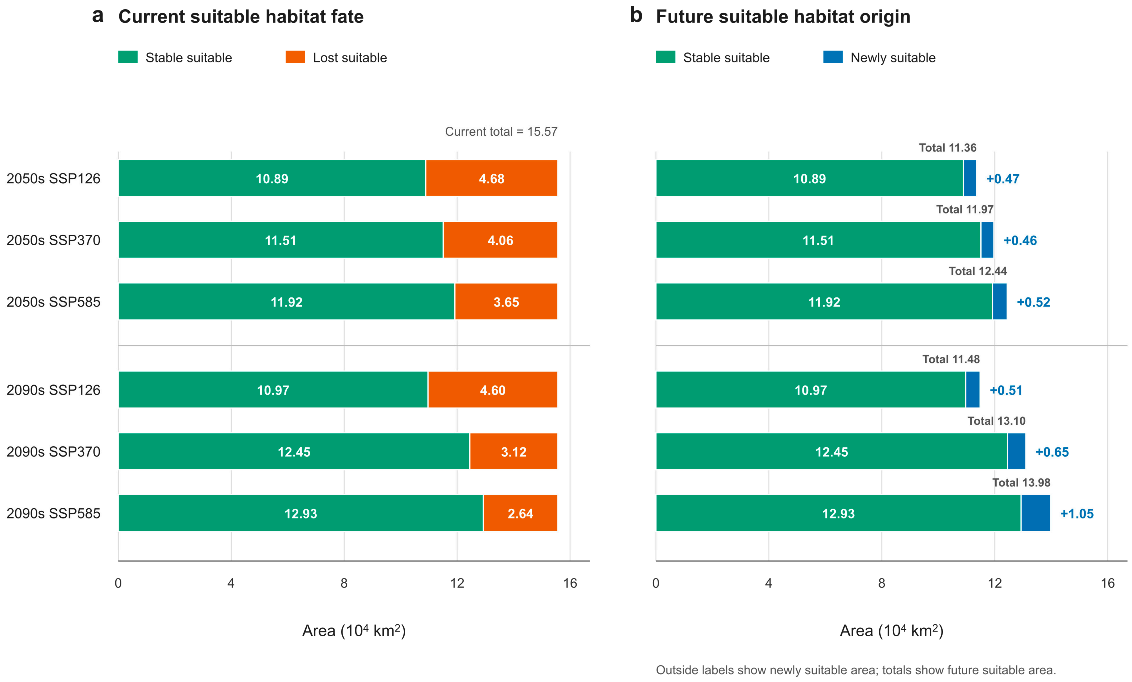

Figure S4. Area composition of stable suitable, lost suitable, and newly suitable habitats under future climate scenarios. V alues indicate area in 10^4 km².

Table S1 Latitude and Longitude of Species Distributions

Voucher No.	Longitude	Latitude	Voucher No.	Longitude	Latitude
MYSF-24-001	101.014425	30.031533	MYSF-24-038	100.239499	30.252447
MYSF-24-005	102.224019	31.908081	MYSF-24-040	100.08471	31.47944
MYSF-24-006	101.563323	30.348821	MYSF-24-041	100.06222	31.47993
MYSF-24-007	101.070147	30.132189	MYSF-24-044	101.25584	30.863028
MYSF-24-009	101.101514	30.974202	MYSF-24-046	101.246325	30.665538
MYSF-24-010	101.956647	30.082331	MYSF-24-049	98.84677	31.14936
MYSF-24-011	100.288024	31.218417	MYSF-24-052	100.23631	31.63292
MYSF-24-015	102.379861	32.744111	MYSF-24-053	100.212868	30.011704
MYSF-24-019	101.917942	29.87915	MYSF-24-054	98.535511	33.004419
MYSF-24-022	101.522975	30.319186	MYSF-24-057	101.5616	32.99087
MYSF-24-026	100.560553	31.363478	MYSF-24-058	97.81	33.4275
MYSF-24-029	100.07709	31.48222	MYSF-24-060	102.115919	31.926311
MYSF-24-034	100.07049	31.48589	MYSF-24-062	98.84799	31.1531
MYSF-24-035	97.744441	33.829442	MYSF-24-063	100.526146	31.501665
MYSF-24-036	99.818333	33.136111	MYSF-24-065	102.831	33.598278
MYSF-24-037	97.79605	33.139189			

Table S2. Environmental predictors retained for model calibration.

Code	Environmental variable	Category	Unit
bio2	Mean diurnal range	Climatic	°C
bio3	Isothermality	Climatic	%
bio4	Temperature seasonality	Climatic	—
bio5	Max temperature of warmest month	Climatic	°C
bio12	Annual precipitation	Climatic	mm
bio15	Precipitation seasonality	Climatic	%
bio17	Precipitation of driest quarter	Climatic	mm
aspect	Aspect	Topographic	degree
slope	Slope	Topographic	degree
t_oc	Topsoil organic carbon	Soil	% weight
t_ph_h2o	Topsoil pH in H_2O	Soil	pH
s_ph_h2o	Subsoil pH in H_2O	Soil	pH

Table S3. Model evaluation metrics of single algorithms and the EMwmean ensemble model.

Algorithm	n	AUC	TSS	Kappa	Status based on mean TSS > 0.75
GAM	20	0.998 ± 0.011	0.991 ± 0.042	0.973 ± 0.122	Yes
XGBOOST	23	0.987 ± 0.037	0.970 ± 0.074	0.809 ± 0.081	Yes
RFd	23	0.992 ± 0.002	0.955 ± 0.014	0.737 ± 0.034	Yes
MARS	23	0.959 ± 0.016	0.887 ± 0.058	0.440 ± 0.065	Yes
GBM	23	0.965 ± 0.015	0.866 ± 0.052	0.514 ± 0.070	Yes
GLM	23	0.919 ± 0.017	0.759 ± 0.072	0.303 ± 0.042	Yes
MAXENT	21	0.918 ± 0.014	0.715 ± 0.038	0.466 ± 0.074	No
MAXNET	23	0.915 ± 0.014	0.713 ± 0.038	0.444 ± 0.056	No
FDA	23	0.892 ± 0.031	0.659 ± 0.076	0.374 ± 0.064	No
CTA	23	0.825 ± 0.070	0.638 ± 0.132	0.167 ± 0.086	No
ANN	10	0.781 ± 0.103	0.582 ± 0.213	0.194 ± 0.105	No
SRE	23	0.611 ± 0.046	0.223 ± 0.091	0.061 ± 0.037	No
EMwmean	1	0.907	0.785	0.625	Final ensemble

Table S4. Current habitat suitability area of A. anserina in the study region.

Suitability class	Suitability threshold	Area (10^4 km^2)	Percentage (%)
Unsuitable habitat	[0, 0.1)	11.65	42.8
Low-suitability habitat	[0.1, 0.3)	7.92	29.1
Moderate-suitability habitat	[0.3, 0.5)	3.97	14.6
High-suitability habitat	[0.5, 1.0]	3.68	13.5
Total suitable habitat	≥ 0.1	15.57	57.2

Table S5. Areas of stable suitable, lost suitable, and newly suitable habitats under future climate scenarios (10^4 km²).

Scenario	Stable suitable	Lost suitable	Newly suitable	Future suitable from overlay
2050s SSP126	10.89	4.68	0.47	11.36
2050s SSP370	11.51	4.06	0.46	11.97
2050s SSP585	11.92	3.65	0.52	12.44
2090s SSP126	10.97	4.60	0.51	11.48
2090s SSP370	12.45	3.12	0.65	13.10
2090s SSP585	12.93	2.64	1.05	13.98

Table S6. Spatial dynamics and centroid migration of suitable habitats of *A. anserina* under future climate scenarios.

**Scenario**	**Centroid longitude**	**Centroid latitude**	**Centroid shift (km)**
Current	100.785	31.440	0.0
2050s SSP126	100.466	31.599	35.0
2050s SSP370	100.579	31.641	29.6
2050s SSP585	100.461	31.527	32.2
2090s SSP126	100.358	31.651	46.8
2090s SSP370	100.578	31.596	26.1
2090s SSP585	100.659	31.450	12.1

Note: Centroid coordinates are given in decimal degrees.

Table S7. Niche overlap indices between current and future climate scenarios.

Scenario	Schoener's D	Niche overlap I
2050s SSP126	0.702	0.796
2050s SSP370	0.635	0.737
2050s SSP585	0.670	0.762
2090s SSP126	0.698	0.781
2090s SSP370	0.573	0.664
2090s SSP585	0.534	0.634
